# Statistical Cluster Analysis of the British Thoracic Society Severe Refractory Asthma Registry: Clinical Outcomes and Phenotype Stability

**DOI:** 10.1371/journal.pone.0102987

**Published:** 2014-07-24

**Authors:** Chris Newby, Liam G. Heaney, Andrew Menzies-Gow, Rob M. Niven, Adel Mansur, Christine Bucknall, Rekha Chaudhuri, John Thompson, Paul Burton, Chris Brightling, on behalf of the British Thoracic Society Severe Refractory Asthma Network

**Affiliations:** 1 Department of Infection, Inflammation and Immunity, Institute for Lung Health, University of Leicester, Leicester, United Kingdom; 2 Department of Health Sciences, University of Leicester, Leicester, United Kingdom; 3 Centre for Infection and Immunity, Queen's University of Belfast, Belfast, United Kingdom; 4 Royal Brompton Hospital, London, United Kingdom; 5 North West Lung Centre, University of Manchester, Manchester, United Kingdom; 6 Severe and Brittle Asthma Unit, Birmingham Heartlands Hospital, Birmingham, United Kingdom; 7 Department of Respiratory Medicine, Stobhill Hospital, Glasgow, United Kingdom; 8 Department of Respiratory Medicine, Division of Immunology, Infection and Inflammation, University of Glasgow and Gartnavel General, Glasgow, United Kingdom; University of Louisville, United States of America

## Abstract

**Background:**

Severe refractory asthma is a heterogeneous disease. We sought to determine statistical clusters from the British Thoracic Society Severe refractory Asthma Registry and to examine cluster-specific outcomes and stability.

**Methods:**

Factor analysis and statistical cluster modelling was undertaken to determine the number of clusters and their membership (N = 349). Cluster-specific outcomes were assessed after a median follow-up of 3 years. A classifier was programmed to determine cluster stability and was validated in an independent cohort of new patients recruited to the registry (n = 245).

**Findings:**

Five clusters were identified. Cluster 1 (34%) were atopic with early onset disease, cluster 2 (21%) were obese with late onset disease, cluster 3 (15%) had the least severe disease, cluster 4 (15%) were the eosinophilic with late onset disease and cluster 5 (15%) had significant fixed airflow obstruction. At follow-up, the proportion of subjects treated with oral corticosteroids increased in all groups with an increase in body mass index. Exacerbation frequency decreased significantly in clusters 1, 2 and 4 and was associated with a significant fall in the peripheral blood eosinophil count in clusters 2 and 4. Stability of cluster membership at follow-up was 52% for the whole group with stability being best in cluster 2 (71%) and worst in cluster 4 (25%). In an independent validation cohort, the classifier identified the same 5 clusters with similar patient distribution and characteristics.

**Interpretation:**

Statistical cluster analysis can identify distinct phenotypes with specific outcomes. Cluster membership can be determined using a classifier, but when treatment is optimised, cluster stability is poor.

## Introduction

Severe refractory asthma is a heterogeneous multi-dimensional disease and is a consequence of a variety of pathophysiological mechanisms driven by complex interactions between the host and environment [1]–[4]. Algorithmic cluster analysis, such as hierarchical and k-means clustering, has been used to determine severe or severe refractory asthma phenotypes in adults [5]–[11] and children [12] and these clusters have demonstrated clinical utility with cluster-specific response to therapy [1]. However, these analyses have several limitations including difficulties in determining the best fitting number of clusters, as they are algorithmic by nature and do not rely on statistical inference to determine this number [13]. More importantly, the longitudinal phenotypic stability of described clusters in a clinical setting has not been previously examined. One challenge is that most approaches to clustering can only be applied to the data at a single time point and thus may derive new and additional clusters at another time point in the same population making the assessment of the repeatability of cluster membership and phenotype stability impossible.

An alternative approach to determine partitioning of multivariate data is to use statistical mixture models [14] and these have been applied in several disciplines such as gene-expression [15], neurophysiology [16], and magnetic resonance imaging [17]. Mixture models are a family of statistical models that determine the best fitting number of clusters or mixtures by comparing models with differing number of mixtures or clusters using model selection criteria to choose the best-fitting model such as the Akaike information criterion [18] and the Bayesian information criterion [19]. These models are less sensitive to outliers and less sensitive to over fitting the data values than k-means and hierarchical clustering [20]. In addition, classifiers that determine cluster membership can be applied to the same population repeatedly and across cohorts to define phenotypic stability.

Here we report in a multi-centre longitudinal observational study of severe refractory asthma the application of statistical clustering, determine phenotypic-specific changes over time and define the consequent affects upon phenotype stability.

## Materials and Methods

### Ethics statement

Written informed consent for data entry and analysis from the multi centres was obtained from all subjects and the study was approved by each centre's local ethics committee. Ethical approval for the British Thoracic Society Difficult Asthma Registry across sites was obtained from the Office for Research Ethics Committees Northern Ireland (number 10/NIR02/37). In Leicester additional ethics was also obtained from the Leicestershire, Northamptonshire and Rutland ethics committee (REC 6307).

### Subjects and protocol

Subjects were recruited from centres contributing data to the British Thoracic Society Severe refractory Asthma Registry [21]–[25]. Subjects were assessed using a standardised protocol that included the recording of demographics, pulmonary function tests, allergy assessment, and standardised blood panel, including peripheral blood eosinophil count and total serum immunoglobuin (IgE). Data was entered into an anonymised, secure, web-based registry hosted by Dendrite Clinical Systems Ltd. Subjects in the first dataset were from 4 centres the Regional Severe refractory Asthma Service, Belfast City Hospital, Institute for Lung Health, Glenfield Hospital, Leicester, Royal Brompton Hospital, London and North West Lung Centre, Manchester. These subjects were assessed at baseline and after a minimum of 1 year follow-up. Subjects were clinically managed in accordance with local and national guidelines to optimise asthma control, and minimize future risk of exacerbations, lung function impairment and side-effects to therapy. The characteristics of these subjects have been described previously [21]–[25]. The severe refractory patients were defined by being on oral steroids 50% of the year or on high dose inhaled steroids plus add on medications either at baseline or follow-up.

The second cross-sectional validation cohort was recruited from 7 centres including the original 4 centres as well as Gartnavel Hospital and Stobhill Hospital in Glasgow, and the Heart of England Hospital, Birmingham. Written informed consent for data entry and analysis was obtained from all subjects and the study was approved by each centre's local ethics committee.

### Statistical analysis

Statistical analysis was carried out in R programming language and SPSS 10.0 for Windows. Multiple imputation algorithms were undertaken to allow for missing values. Variables were selected for use in the multiple imputation analyses that satisfied several conditions; they had less than 30% missing data and the missing data was either classified as missing completely at random or missing at random [26]. In order to determine which variables were deemed missing at random and not missing at random we used prior knowledge from multiple clinicians to determine if data missing was due to patient severity or due to random chance of missing information in medical records. Severity is the key mechanism for informative missing-ness for severe asthma as if patients are too severe some of the measurements are contraindicated. The cases in variables that were not missing due to severity of asthma were included in the multiple imputation algorithm.

This reduced the total number of variables to 23 variables on which multiple imputation was carried out, see supplement for a list of variables used and for more details on multiple imputation. Multiple imputation can be carried out for up to 70% missing data in some cases if the variables are missing completely at random, missing at random or if there is no bias from missing samples [26]. The missing variables are predicted using regression equations and 5 missing values are added for each variable. This methodology obtains less biased parameter estimates than removing the patients with missing values or imputing with the mean [26]. The mi package in R was used for the implementation of the missing data prediction as it allowed for convergence of the model to be checked. Convergence is checked for both model parameters and data before deriving the missing values to obtain good practices and good predictions for missing values see online supplement.

Factor analysis was carried out in SPSS using principal component methodology with varimax rotation to determine factor scores and eigen values for each factor. We use factor analysis to determine underlying clinical processes. The underlying processes can be thought of as the independent structure of the data. The factor analysis was carried out using the available non-categorical variables. The treatment variables oral steroid dose and Beclomethasone Dipropionate (BDP) equivalent dose inhaled corticosteroids were transformed into a ranked treatment variable before factor analysis, with patients with the highest inhaled steroid treatment and highest oral steroid dose having the highest rank. The underlying factors from the factor analysis were saved as variables describing independent asthma pathology using the derived factor scores. The factor scores were then used as input variables for the cluster analysis to determine sub-groups within the differing asthma pathology.

The clustering was carried out using a two way cluster/mixture analysis [27], [28] model that compares models for 1 to 15 clusters using the Bayesian information criterion. The input variables for the cluster analysis were the output latent factors from the previous factor analysis. A cluster analysis was carried out for each of the 5 imputed datasets with 5 cluster membership as outcomes. The 5 cluster membership were combined using the same two-way cluster algorithm but for categorical data to obtain a global average cluster membership, for the many steps of the multiple imputation cluster analysis please see Figure E1 in File S1. Comparisons between clusters were made using the most appropriate test: one-way analysis of variance (ANOVA) for normally distributed data, χ^2^ -test for proportional data and Kruskal-Wallis test for non-normally distributed variables. The non-missing data for each variable was used when testing. The follow up data was analysed by applying paired t-tests or Wilcoxon matched pair signed rank tests as appropriate to determine differences over time for each cluster, general estimating equations (GEE) models were also used but not presented here.

After cluster membership is determined a classifier can be created. A classifier uses cluster specific statistics such as the mean and standard deviation of key variables to determine cluster membership for new patients, it is a stand-alone algorithm that unlike the cluster analysis does not require the rest of the data to predict cluster membership. The clinical variables used as inputs depend on their predictive accuracy of cluster membership with the best predictors being kept into the classifier. A function in R was programmed to classify the patients into one of the five clusters, based on the specific cluster multivariate parameters that were found in the cluster analysis. Every cluster has a unique multivariate distribution based on a subset of input parameters. A patient is classified as belonging to the cluster with the most likely cluster multivariate characteristics for that patient, without the need of the rest of the data to determine cluster membership. Once classified the patients (supervised) cluster classification was compared with their original (unsupervised) cluster classification to determine percentage accuracy at baseline and stability at follow-up. The classifier was applied to a second dataset to determine if the clusters were in similar proportions and had similar characteristics to the original dataset. A p value of <0.05 was taken as the threshold of statistical significance. For further details of the classifier see online supplement.

## Results

Three-hundred and forty-nine subjects from 4 centres were included in the baseline analysis. The criteria for selection of the variables used in the factor analysis were thus: Variables had to have less than 30% missing data in the original cohort and were also non-categorical. Five factors were found for the continuous variables using the Kaiser criteria for selection of the number of factors. Five factors were found for the continuous variables, these factors or aspects can be described as representing airflow obstruction, exacerbation frequency, IgE/body mass index (BMI), treatment scaling and peripheral blood eosinophilia (Table 1). These five derived factor variables were used as input variables in to the cluster analysis. The optimal number of clusters that were identified for each multiple imputed dataset is shown in Table E1 in File S1 with the BIC for each cluster model and imputed dataset in Figure E2 in File S1. The cluster memberships returned were all very similar with very similar number of clusters, although as previously discussed removal of missing data would give a more concrete cluster membership and number of clusters. Only using patients that had no missing data would create a selection bias.

**Table 1:** Factor loading for continuous variables from first imputed data set, other factor analysis on the other 4 imputed datasets showed similar structure and factor loading.

	Continuous Factors				
	1	2	3	4	5
	Airflow Obstruction	Exacerbation	IgE/Obesity	Treatment scaling	Blood Eosinophilia
Cumulative Percentage of variance explained by factors	21.3%	37.5%	51.4%	62.6%	72.1%
BMI	.050	.205	.651	.346	.005
Pre-bronchodilator FEV_1_ % predicted	.990	−.004	−.012	−.070	−.031
Pre-bronchodilator FVC %predicted	.784	−.036	−.338	.123	.139
Pre-bronchodilator FEV_1_/FVC	.724	.109	.296	−.195	−.196
Blood eosinophils ×10^9^/L	−.067	.166	−.155	−.050	.819
Total IgEkU/L	.070	.068	−.621	.058	.071
Exacerbation frequency (number of rescue oral steroid courses in last year)	.052	.796	−.063	.247	.092
Number of primary care visits in last year	.013	.759	.069	.350	.010
Treatment	−.090	−.029	.067	.847	−.077
Age at onset of symptoms	.043	−.225	.519	−.071	.572

Membership was taken from each imputed dataset and used as a variable to carry out latent class analysis to determine mean cluster membership. A five cluster model best described the dataset. Their characteristics are as shown Table 2.

**Table 2:** Clinical characteristics of the original BTS severe refractory asthma clusters.

	All (N = 349)	Cluster 1 (N = 117; 34%) ‘Early onset, atopic’	Cluster 2 (N = 72; 21%) ‘Obese, late onset’	Cluster 3 (N = 52; 15%) ‘normal lung function, least severe asthma’	Cluster 4 (N = 54; 15%) ‘late onset, eosinophilic’	Cluster 5 (N = 54, 15%) ‘Airflow obstruction’	p-value
Gender, (% Male) (N = 349)*	36.4	31.6	40.3	23.1	48.1	42.6	0.042
Age At Baseline (years) (n = 345)^‡^	21 (18)	40 (13)	47 (10)	43 (15)	49 (16)	50 (12)	<0.001
Age At Onset Of Symptoms (years)^‡^	26 (19)	13 (13)	39 (14)	27 (19)	40 (18)	25 (20)	<0.001
BMI (N = 342)^‡^	29 (6)	27.9(4.43)	36(6.2)	27(6.39)	26.3(5.47)	28(5.06)	<0.001
Pack year history (for whole population) (N = 349)^†^	10 (15)	4(9.5)	12(11.5)	10(14)	17.5(25)	15(10)	0.008
HAD Anxiety Score (N = 160)^†^	8 (4)	9(7.5)	9(4)	4.5(4)	8(5.5)	6(2.75)	0.001
HAD Depression Score (N = 160)^†^	6 (4)	6(7)	8(5)	2.5(3)	4.5(3.75)	4(5.5)	0.002
PC20 Methacholine challenge (N = 20)^†^	1 (2)	2 (2)	2 (1)	1 (3)	1 (1)	1 (1)	0.704
Exhaled No flow rate50 (N = 124)^†^	37 (51)	34 (45)	51 (18)	25 (29)	55 (98)	56 (89)	0.111
Atopy (% Yes) (N = 346)*	58.4	68.7	61.1	44.2	53.7	50.9	0.023
Total IgE blood count, kU/l (N = 319)^‡^	303 (612)	322(361)	151(255)	162(186)	638(1360)	267(253)	<0.001
Perennial rhinitis (% Yes) (N = 340)*	29.1	36	28	19	35	20	0.088
Seasonal rhinitis (% Yes) (N = 340)*	37.9	48	38	33	30	31	0.093
Eczema (% Yes) (N = 341)*	27.9	41	28	19	17	20	0.003
Polyps (% Yes) (N = 340)*	13.5	12	13	12	20	14	0.637
Prior nasal surgery (% Yes) (N = 340)*	15.0	20	13	9.6	17	12	0.423
Reflux history (% Yes) (N = 340)*	42.6	51	47	27	32	46	0.014
Oral steroids (Yes %) (N = 347)*	40.9	51	56	6	17	57	<0.001
Oral steroid dose (mg) (N = 342)^†^	15 (10)	15(10)	15(10)	8(5)	15(10)	10(10)	0.153
BDP equivalent inhaled steroid dose (mcg) (N = 333)^‡^	1853 (976)	2060(995)	2100(1220)	1150(633)	1770(711)	1820(720)	<0.001
Blood Eosinophil count 10^9^/l (N = 327)^†^	0.30 (0.49)	0.28 (0.41)	0.32 (0.34)	0.20 (0.27)	0.87(1.02)	0.21(0.40)	<0.001
Rescue steroid courses in last year (n) (N = 322)^†^	4 (4)	5(4.5)	5(3)	1(3)	4(4)	0(2)	<0.001
Hospital Admission last year (n) (N = 344)^‡^	1 (2)	1.8 (2.6)	1.4(2.2)	0.8(1.3)	1.2 (1.9)	0.4 (0.8)	<0.001
ITU admissions in last year (N = 345)^‡^	0 (1)	0.8 (1.9)	0.3 (0.7)	0.1 (0.3)	0.1(0.5)	0.2 (0.6)	<0.001
ITU admission ever (% yes) (N = 345)*	18.3	31.9	16.7	7.7	9.6	9.4	<0.001
Pre-bronchodilator FEV_1_ (L) (N = 338)^‡^	1.95 (0.82)	1.79(0.731)	2.25(0.649)	2.52(0.847)	1.75(0.881)	1.55(0.743)	<0.001
Pre-bronchodilator FEV_1_ Predicted (%) (N = 330)^‡^	66 (24)	58.8(21.2)	77(19.7)	88.4(21.1)	58.8(20.3)	52.8(18.9)	<0.001
FEV_1_/FVC Pre Bronchodilator (%) (N = 329)^‡^	63 (15)	58.1(14.8)	72.5(10.9)	76.6(9.82)	57.5(12.3)	53.7(12.3)	<0.001
Post-bronchodilator FEV_1_ response (%) (N = 239)	18.5 (23.3)	24.8(28.5)	12.9 (12.0)	11.3(15.0)	19.2(30.5)	18.8(15.8)	0.002

Data represented as ^‡^Mean (SD) tested with ANOVA test, ^†^Median (IQR) tested with Kruskal-Wallis test, *(%) tested with Chi squared test.

Cluster 1 comprised 34% of the cohort; had early onset, atopic asthma with the highest intensive care (ITU) admissions, high exacerbation frequency, half the subjects receiving regular systemic corticosteroid therapy, and poor lung function but with the greatest bronchodilator reversibility. Cluster 2 comprised 21% of the cohort; was an obese, late onset group with frequent exacerbations, over half the subjects receiving regular systemic corticosteroid therapy, near normal lung function and the highest depression score. The remaining three clusters comprised 15% each of the overall population. Cluster 3 subjects were mainly non-atopic with normal lung function, the fewest number of subjects receiving systemic corticosteroid therapy and infrequent exacerbations. Cluster 4 was a late onset group, with a markedly elevated peripheral blood eosinophilia and frequent exacerbations. Cluster 5 had the worst lung function and the highest proportion of subjects receiving systemic corticosteroid therapy, but had the least frequent exacerbations. The proportions of subjects in each cluster varied between centres Table E2 in File S1.

**Figure 1:**
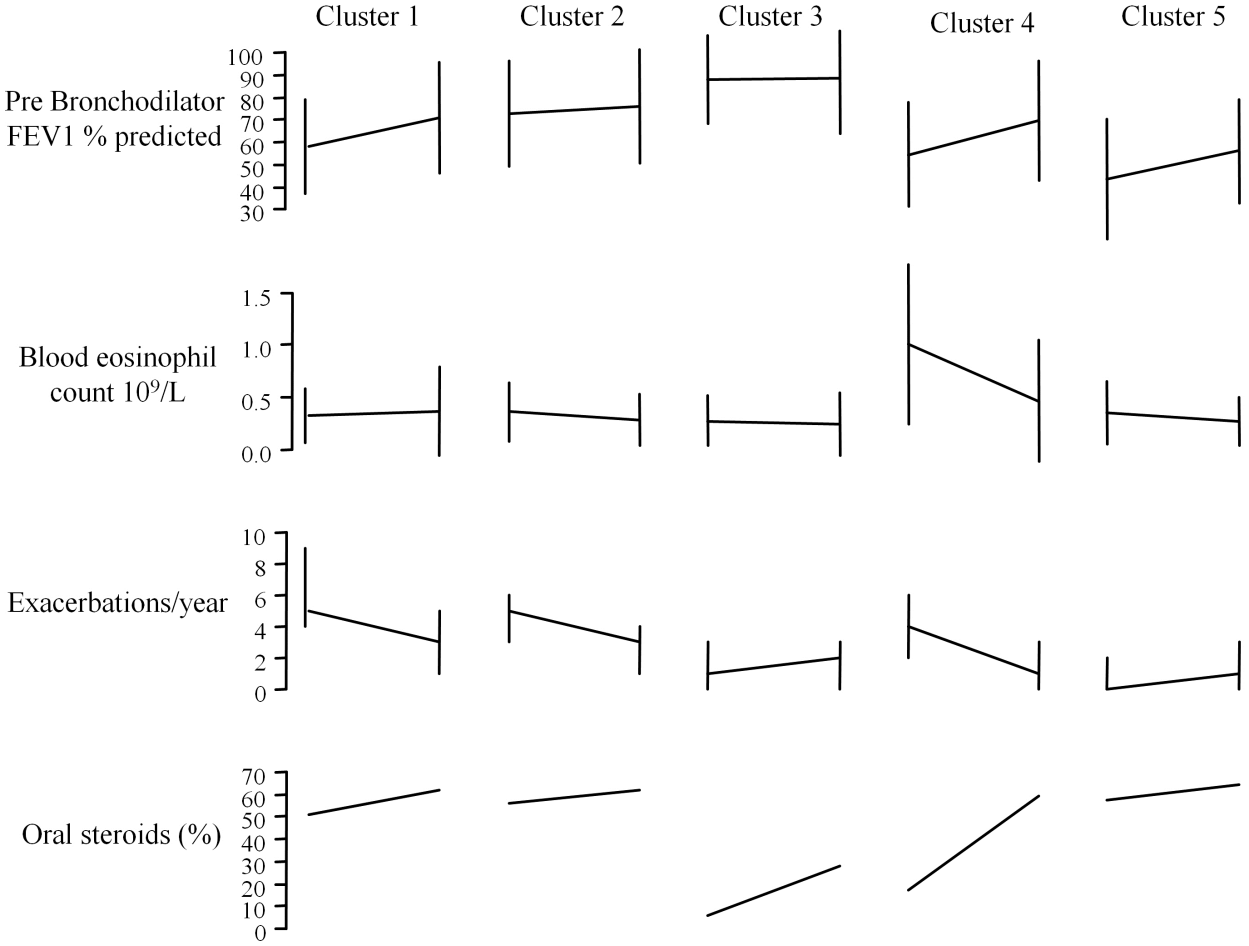
Differences in clinical outcomes between baseline and follow-up for each cluster. Pre Bronchodilator FEV1 % predicted and Blood eosinophil count statistics are Mean (+/−SD). Exacerbation frequency outcomes are median (IQR) and percentage on oral steroids are percentages.

Subjects were followed up for a median (interquartile range) 3.1 (1.9 to 5.5) years. The cluster-specific changes in clinical outcomes at follow-up are as shown (Table 3 and Figure 1), GEE models were also carried out and showed similar effects for the variables but are not presented here. At follow-up the proportion of subjects treated with oral corticosteroids increased in all groups with statistically significant increases for clusters 3 and 4. This was coupled with a concomitant increase in BMI, which was statistically significant in all but cluster 2. Clusters 1, 4 and 5 had statistical improvement in forced expiratory volume in 1 second (FEV_1_) Mean (SD) 0.28 (0.64), 0.28 (0.68), 0.20 (0.54) respectively. There was a statistically significant decrease in exacerbation frequency in clusters 1, 2 and 4 median (IQR) −2 (−6,−0), −2 (−4, 1), −2 (−5, 0) respectively with an associated improvement in the Hospital Anxiety and Depression (HAD) score in clusters 1 for anxiety mean (SD) −1.2 (3.1) and 2 for depression mean (SD)−2.1 (3.5). The peripheral blood eosinophil counts in clusters 2 and 4 mean (SD), −0.08 (0.27), −0.53 (0.88) respectively.

**Table 3:** Cluster specific differences over time for clinically significant outcomes of clusters.

	Cluster 1 ‘Early onset, atopic’		Cluster 2 ‘Obese, late onset’		Cluster 3 ‘normal lung function least severe asthma’		Cluster 4 ‘late onset, eosinophilic’		Cluster 5 ‘Airflow obstruction’		p-value difference between groups
	Difference	P-value	Difference	P-value	Difference	P-value	Difference	P-value	Difference	P-value	
BMI*	1.2 (2.6)	<0.001	0.2(3.0)	0.617	1.6(3.4)	0.004	1.4(2.3)	<0.001	0.8(2.3)	0.017	0.051
Pre bronchodilator FEV_1_^‡^	0.28(0.64)	<0.001	−0.01(0.56)	0.943	−0.02(0.43)	0.754	0.28(0.68)	0.008	0.20(0.54)	0.031	0.011
Pre bronchodilator FEV_1_ % predicted^‡^	11.3 (21.9)	<0.001	2.67 (18.7)	0.275	0.5 (15.5)	0.848	12.8 (25.0)	0.002	10.8 (19.1)	0.001	0.011
Blood Eosinophil count, ×10^9^/l^‡^	−0.03(0.52)	0.575	−0.08(0.27)	0.046	0.01(0.37)	0.941	−0.53(0.88)	<0.001	−0.09(0.27)	0.09	<0.001
BDP equivalent (mcg/day)^‡^	−64(989)	0.495	−229(1205)	0.121	384(884)	0.005	10.6(971.3)	0.937	−2(852)	0.987	0.032
% on steroids at follow up*	61.5%	0.108	62.0%	0.648	28.0%	0.013	59.3%	<0.001	64.2%	0.344	NA
Oral steroid dose (mg)^†^	−0.4 (16.1)	0.868	0.9(12.2)	0.679	0(0)	1.0	5.3(21.3)	0.535	0.9(10.9)	0.670	0.906
HAD Anxiety Score^‡^	−1.2 (3.1)	0.044	0.0(4.6)	1.00	0.3(2.2)	0.704	−1.3(3.6)	0.194	−1.3(2.7)	0.169	0.557
HAD Depression Score^‡^	−1.0(4.4)	0.226	−2.1(3.5)	0.020	0.18(3.1)	0.849	−2.07(5.0)	0.131	−0.7(2.7)	0.486	0.548
Rescue steroid courses in last year (n)^†^	−2 (−6 to 0)	<0.001	−2 (−4 to 1)	0.004	0.5 (−2 to 2)	0.903	−2 (−5 to 0)	<0.001	1 (0 to 3)	0.027	<0.001

Data represented as ^‡^Mean (SD) tested with paired t-test for each cluster, ^†^Median (IQR) tested with paired sample Wilcoxon signed rank test, * (%) tested with McNemar's test.

The classifier produced 79% correct cluster membership when compared with original cluster membership at baseline (Table 4) with Cluster 2 being the best predicted and Clusters 1 and 4 being predicted well. The classifier was used on data from the follow-up time point to determine if clusters remained consistent over time. The classification at the next time point compared to the baseline clustering was 52% (Table 5). Patients that were originally in cluster 2, the obese asthmatic group, were the most consistent over time. The largest changes were that a substantial proportion of the patients in clusters 1, 4, and 5 moved into clusters 2 and 3 at follow up reflecting the changes in lung function, peripheral blood eosinophilia and exacerbation frequency.

**Table 4:** Cluster and Classification membership compared at baseline.

	Predicted cluster classification for baseline data				
Baseline cluster	Cluster 1 ‘Early onset, atopic’	Cluster 2 ‘Obese, late onset’	Cluster 3 ‘normal lung function least severe asthma’	Cluster 4 ‘late onset, eosinophilic’	Cluster 5 ‘Airflow obstruction’
1	**80.0**	7.1	5.9	3.5	3.5
2	4.4	**88.9**	2.2	4.4	0.0
3	2.6	10.3	**74.4**	2.6	10.3
4	5.3	2.6	2.6	**86.8**	2.6
5	16.7	10.0	13.3	0.0	**60.0**

**Table 5:** Cluster memberships at baseline compared to classification membership at follow up.

	Predicted clusters from year follow up data				
Baseline cluster	Cluster 1 ‘Early onset, atopic’	Cluster 2 ‘Obese, late onset’	Cluster 3 ‘normal lung function least severe asthma’	Cluster 4 ‘late onset, eosinophilic’	Cluster 5 ‘Airflow obstruction’
1	**52.2**	15.2	10.9	13.0	8.7
2	3.2	**71.0**	12.9	3.2	9.7
3	6.7	40.0	**46.7**	6.7	0.0
4	25.0	15.0	15.0	**25.0**	20.0
5	12.5	18.8	18.8	0.0	**50.0**

The classifier was also used to classify data on the new dataset of 245 severe refractory asthma patients presenting to seven specialist centres including the 4 original centres. These new patients received a cluster annotation. The clusters were in similar proportions in the new dataset and shared similar properties to the original cohort (Table 6).The proportions of subjects in each cluster were different across the centres (Table E2 in File S1). The cluster specific variables were tested between each dataset to determine cluster similarities across the two datasets (Table E3 in File S1).

**Table 6:** Clinical characteristics for new BTSsevere refractory asthma dataset using the classifier.

	All (N = 245)	Cluster 1 (N = 90; 37%) ‘Early onset, atopic’	Cluster 2 (N = 68; 28%) ‘Obese, late onset’	Cluster 3 (N = 35; 14%) ‘normal lung function least severe asthma’	Cluster 4 (N = 32; 13%) ‘late onset, eosinophilic’	Cluster 5 (N = 20; 8%) ‘Airflow obstruction’	p-value
Gender, (% Male)*	36	33	32	26	47	60	0.115
Age At Baseline (years) ^‡^	44 (14)	40.2(13.7)	48.7(12.3)	44.5(15)	49(14.6)	45.8(13.8)	0.003
Age At Onset Of Symptoms (years)^‡^	23 (18)	10.2(9.97)	33.5(15.6)	27.3(19.1)	34.5(16.5)	23(21.4)	<0.001
BMI^‡^	30.2 (6.7)	28.6(5.34)	36(6.74)	27.6(6.21)	26(4.33)	28.8(4.34)	<0.001
Pack year history (for whole population)^†^	25 (49)	9(21)	19(28.5)	6.5(8.5)	4(8.5)	17.5(51.5)	0.019
HAD Anxiety Score^‡^	9 (5)	8(4.77)	9.86(4.92)	8.88(5.1)	7.72(4.59)	7.27(5.68)	0.221
HAD Depression Score^‡^	7 (4)	6.29(4.26)	8.08(4.54)	6.46(5.07)	6.38(3.68)	6.47(4.27)	0.263
Atopy (% Yes) *	65	72	61	66	65	59	0.793
Total IgE blood count kU/l^‡^	498 (2023)	557(1010)	229(262)	102(124)	370(404)	576(797)	<0.001
Perennial rhinitis (% yes)*	40	43	34	43	45	24	0.629
Seasonal rhinitis (% yes)*	40	42	38	43	44	35	0.897
Eczema (% yes)*	28	36	21	26	28	25	0.479
Polyps (% yes)*	16	14	15	23	22	10	0.705
Prior nasal surgery (% yes)*	17	16	16	17	22	20	0.943
Reflux history (% yes)*	58	51	66	63	56	55	0.530
Oral steroids (yes %)*	36	67	40	27	22	50	0.168
Oral steroid dose (mg)^†^	20 (41)	10(15)	12(10)	15(25)	10(4.5)	11(17.5)	0.160
BDP equivalent inhaled steroid dose (mcg)^‡^	2080 (1029)	2130(873)	2280(1260)	1780(747)	1870(819)	2140(1520)	0.075
Blood Eosinophil count 10^9^/l^†^	0.41 (0.49)	0.3(0.268)	0.205(0.252)	0.17(0.205)	1.03(0.59)	0.24(0.275)	<0.001
Rescue steroid courses in last year (n)^†^	6 (4)	6(4.8)	7(5)	3(5)	5(3.5)	1(1.3)	<0.001
Hospital Admission last year (n)^‡^	1 (2)	1.3(2.2)	1.4(2.3)	1(1.8)	1.4 (2.1)	0.4 (0.7)	0.146
ITU admissions in last year^‡^	0 (2)	0.7 (2.3)	0.4 (1.4)	0.03 (0.2)	0.3 (0.8)	0.6 (1.1)	0.016
ITU admission ever (% yes)	22	27	22	3	16	30	0.013
Pre-bronchodilator FEV_1_ (L)^‡^	2.10 0.87)	2.06(0.889)	2.15(0.809)	2.81(0.941)	1.78(0.778)	1.58(0.501)	<0.001
Pre-bronchodilator FEV_1_ Predicted (%)^‡^	73 (25)	68.7(24.7)	77.3(23.9)	98.8(19.6)	62.5(21.9)	50.6(15.6)	<0.001
FEV_1_/FVC Pre Bronchodilator (%)^‡^	65 (15)	61.4(15.8)	67(16)	76.1(8.77)	62.4(12.4)	57.2(13.1)	<0.001
Post-bronchodilator FEV_1_ response (%)^‡^	0.19 (0.22)	0.19 (0.20)	0.19 (0.16)	0.16 (0.14)	0.15 (0.14)	0.38 (0.46)	0.044

Data represented as ^‡^Mean (SD) tested with ANOVA test, ^†^Median (IQR) tested with Kruskal-Wallis test, *(%) tested with Chi squared test.

## Discussion/Conclusions

We describe here for the first time the application of statistical clustering in a multi-centre longitudinal observational study of the British Thoracic Society Severe refractory Asthma Registry. To date this is the largest group of severe refractory asthma patients to be included in a cluster analysis. Five clusters were identified and using a classifier we have validated these clusters in an independent cohort of patients submitted to the Registry. We have determined phenotypic-specific changes over time and applied the classifier to report for the first time cluster membership stability over time, when treatment is optimised. Stability of cluster membership at follow-up was 52% for the whole group ranging from 25–71% within clusters. Therefore, statistical cluster analysis can identify distinct phenotypes with specific outcomes, but the stability of cluster membership is poor.

The clusters were obtained by carrying out statistical mixture models, where a different number of clusters are tried iteratively and compared using the model fitting criteria Akaike information criterion and the Bayesian information criterion to obtain the statistically best fitting number of clusters. This clustering method allows the number of clusters to be obtained objectively rather than relying on graphs or expert opinion. The five clusters identified were Cluster 1 (34%) the most atopic with early onset disease, cluster 2 (21%) obese with late onset disease, cluster 3 (15%) least severe disease, cluster 4 (15%) the most eosinophilic with late onset disease and cluster 5 (15%) severe airflow obstruction. To further validate these clusters a classifier was created to predict cluster membership for the original dataset and was applied to a new dataset of independent patients recruited to the British Thoracic Society Severe refractory Asthma Registry. This classifier was able to assign cluster membership with 79% accuracy. The 5 clusters in the validation group were of similar proportions and had very similar clinical characteristics.

It is worth noting that all conclusions from the data are found using the cluster and classifier methodology. We present an un-biased statistical cluster analysis technique which selects the number of clusters based on the fit if the data. Bias could come from the choice of variables to include in the cluster analysis we chose asthma related-variables that had minimal missing values. The classifier plays a crucial role in monitoring asthma stability, we used the classifier that best derived the clusters from the data to obtain the best results for all clusters but the conclusions are limited to the accuracy of the classifier which was good in this case.

Although cluster instability could be due to many factor such as cluster methodology, classifier accuracy, underlying disease variability or response to treatment. The cluster instability we found was associated with the clinical characteristics in patients in clusters significantly changing over follow up. This significant change caused them to look like other cluster characteristics found at baseline, suggesting that the cause of cluster instability is due to either the disease variability or response to treatment and not due to statistical methodology.

Our confidence that these clusters are robust is also derived from comparison with other clusters described in the literature. The first multi-centre cluster analysis of asthma undertaken in North America the Severe Asthma Research Network (SARP) [6] also found 5 clusters, but only 3 of these represented patients with severe disease. There were similarities with our clusters with the identification of an obese female predominant cluster, a cluster with moderate airflow obstruction and another with severe airflow obstruction. In the first cluster analysis of severe asthma undertaken in a single-centre study in Leicester, UK Haldar *et al* [5] also described 5 clusters annotated as ‘early-onset atopic’, ‘obese female’, ‘benign asthma’, ‘inflammation predominant’ and ‘early symptom predominant’, which was replicated in an independent group of patients from the same centre [7]. The first 4 clusters match extremely well with the clusters described here in this multi-centre study. Spirometry was not included in the cluster analysis in the Haldar *et al* study and we did not have data for the asthma control questionnaire in sufficient subjects to include in the analysis and this is likely to explain the small differences in cluster characteristics between these studies. Taken together these studies do suggest that cross-sectionally these cluster phenotypes are robust across western European and North American populations of refractory/severe asthma, although importantly the proportions vary between centres which is likely a reflection of referral patterns, but possibly due to real geographical differences. Interestingly, recent data from a multi-centre Korean study identified four refractory asthma clusters [8] that were mostly different to our study or the previously reported UK and USA studies suggesting possible influences of geography and ethnicity.

The potential utility of the identification of refractory/severe asthma clusters is whether they represent distinct endotypes with different underlying aetiology and immunopathogenesis and whether they predict responses to therapy and natural history ([1], [3], and [4]). We did not investigate the potential immunological or pathophysiological mechanisms for the clusters and this warrants further investigation. The patients were managed clinically and follow-up data was available at a single time point with a median time of follow-up of 3 years. At follow-up the proportion of subjects treated with oral corticosteroids increased in all groups with significant increases for cluster 3 and 4. This was coupled with a concomitant increase in BMI, which was significant in all but cluster 2. Clusters 1, 4 and 5 had statistical improvement in FEV_1_, but this change was not closely associated with changes in therapy or peripheral blood eosinophil count. The disconnect between eosinophilic inflammation and lung function is well described [4], but the inconsistency between improvement in lung function and increased therapy may reflect changes in adherence to therapy during the study, which was not formally assessed across centres, or perhaps in part due to regression towards the mean in the clusters with more impaired lung function. Exacerbation frequency decreased significantly in clusters 1, 2, and 4 and was associated with a significant fall in the peripheral blood eosinophil count in clusters 2 and 4 and a small non-significant decrease in cluster 1. In contrast, in cluster 5 there was a fall in the eosinophil count with a small increase in exacerbation frequency, but this small increase in exacerbation frequency is perhaps more likely to reflect regression to the mean than a clinically important difference. An association between eosinophilic inflammation and exacerbations is consistent with previous observations that eosinophilic inflammation is a good predictor of response to corticosteroid therapy [29] and interleukin-5 monoclonal antibody therapy [30], [31], and that the reduction in exacerbation frequency is also related to the reduction in peripheral blood eosinophilia.

Cluster membership stability was assessed at follow-up using the classifier. This was 52% for the whole group with stability being best in cluster 2 (71%) and worst in cluster 4 (25%). The largest changes were that a substantial proportion of the patients in clusters 1, 4, and 5 moved into clusters 2 and 3 at follow up reflecting improvements in lung function, peripheral blood eosinophilia, and exacerbation frequency with increasing obesity. The instability of the cluster membership is perhaps unsurprising in a disease characterised by variability over time and in a real world study in which interventions are instigated as per clinical guidelines rather than in a controlled study. This is not problematic when the instability of the phenotype is used to direct therapy at the time of an assessment for example with the use of a sputum eosinophilia to titrate corticosteroid therapy [32]. However, if emerging phenotype-specific therapies [33] require knowledge of phenotype stability within a patient this will require an understanding of the dynamics of a patient's phenotype using repeated assessments over time and possibly in response to exacerbation events and therapeutic interventions.

One major criticism of this study is that although several domains of the disease were assessed the asthma control and quality of life measures were not recorded systematically in all centres and therefore were insufficient for inclusion in this analysis. Changes in asthma control have a substantial impact upon morbidity and clinical decisions and their absence here does limit interpretation of why therapies were changed in some of the clusters. There is increasing recognition that poor adherence is common in severe refractory asthma, although its identification and management is complex [34]. The disconnect between some of the clinical outcomes and change in therapy would have been better informed in light of adherence rates in the clusters. Importantly, cluster membership is defined by what is measurable at the time of the study. In order to unlock other possible sub-groups or verify cluster immunopathogenesis data at other scales of the disease for example from tissue samples, sputum, protein or transcriptomic signatures from the host and analysis of environmental exposure such as the microbiome may shed more light upon the mechanisms driving the clusters. Although this report is amongst the largest groups of severe asthmatics studied our findings still require further validation in larger populations across different geographical locations with different health care systems. In particular, we found differences across centres in a single country and the reasons for this variability needs to be investigated. Additionally, the follow-up was limited to a single assessment and further work more carefully examining the dynamics of the severe refractory asthma phenotypes are required.

In conclusion, statistical clustering of the British Thoracic Society Severe refractory Asthma Registry identified 5 clusters that were validated in an independent dataset and had many similarities with earlier algorithmic cluster analyses. Follow-up of these clusters demonstrated phenotype-specific outcomes and consequently considerable cluster instability overtime supporting the view that severe refractory asthma is a homeokinetic as well as heterogeneous condition. Understanding this heterogeneity over time remains an important challenge to advance stratified medicine.

## Supporting Information

File S1 Supplementary information for main manuscript “Statistical cluster analysis of the British Thoracic Society severe refractory asthma registry: clinical outcomes and phenotype stability.”
(DOCX)
