# Supplementary material for: Statistical Cluster Analysis of the British Thoracic Society Severe Refractory Asthma Registry: Clinical Outcomes and Phenotype Stability
Source: PLoS One. 2014 Jul 24;9(7):e102987. doi: 10.1371/journal.pone.0102987 (PMC4109965; doi:10.1371/journal.pone.0102987)
Supplement: File S1 — Supplementary information for main manuscript “Statistical cluster analysis of the British Thoracic Society severe refractory asthma registry: clinical outcomes and phenotype stability.” (DOCX) [file pone.0102987.s001.docx]

**Online supplement**

**Statistical cluster analysis of the British Thoracic Society difficult asthma registry: clinical outcomes and phenotype stability**

Chris Newby^1, 2^, Liam G Heaney^3^, Andrew Menzies-Gow^4^, Rob M Niven^5^, Adel Mansur^6^, Christine Bucknall^7^, Rekha Chaduri^8^, John Thompson^2^, Paul Burton^2^, and Chris Brightling^1^on behalf of the British Thoracic Society Difficult Asthma Network

^1^Department of Infection, Inflammation and Immunity, Institute for Lung Health, University of Leicester, UK.^2^Department of Health Sciences, University of Leicester, UK.^3^Centre for Infection and Immunity, Queen's University of Belfast, UK.^4^Royal Brompton Hospital, London, UK.^5^North West Lung Centre, University of Manchester, Manchester, UK.^6^Severe and Brittle Asthma Unit, Birmingham Heartlands Hospital.^7^Department of Respiratory Medicine, Royal Infirmary, Glasgow, UK.^8^Department of Respiratory Medicine, Division of Immunology, Infection and Inflammation, University of Glasgow and Gartnavel General, Glasgow, UK.

**Correspondence to:**

Professor CE Brightling

Institute for Lung Health

Clinical Sciences Wing

University Hospitals of Leicester

Leicester, LE3 9QP, UK

Tel: 0044 116 258 3998, Fax: 0044 116 258 3950

E-mail: [ceb17@le.ac.uk](mailto:ceb17@le.ac.uk)

**Overview of Statistical Analysis used in Paper**

**Fig E1 demonstrates the steps in the analysis to determine an overall cluster membership.**

The dataset had several missing values. Variables with 30% or less missing data were included in the analysis plan. The MI [1] package in R [2] was used to carry out multiple imputation over the dataset this used a multiple iterative regression imputation technique [3] to create 5 datasets with differing values imputed for their missing values. Factor analysis was carried out using SPSS [4] on each dataset, see fig E1, to determine if the patterns of correlation between variables were preserved in the 5 new datasets and to describe the data in each dataset as a smaller amount of independent variables known as factors or components. The principle components algorithm with varimax rotation was used in SPSS for implementing the factor analysis. Rather than using a selection of variables into the cluster analysis we use the derived factors as independent inputs into the cluster analysis as the clustering method used here needed independent variables as inputs and these factors represent the underlying processes of the severe asthmatics. The cluster analysis used was the two way cluster analysis in SPSS [5, 6]. The independent factors from each of the five datasets were used for five cluster analysis, creating five cluster memberships, see fig E1. The number of clusters was selected by selecting the cluster solution with the best (lowest) corresponding BIC [8] for each dataset, see table E1 and fig E2. The 5 cluster memberships were then merged using the two way cluster analysis for categorical data in SPSS [5, 6], see Fig E1 again.

**Variables used for multiple imputations**

1. Gender, 2) body mass index (BMI), 3) smoking status, 4) atopy (Yes/No), 5) unscheduled visits to GP in last 12 months, 6) intensive care unit (ITU) visits in last 12 months, 7) hospital admissions in last 12 months, 8) perennial rhinitis (Yes/No), 9) seasonal rhinitis (Yes/No), 10) eczema (Yes/No), 11) Nasal Polyps (Yes/No), 12) Reflux history (Yes/No), 13) pre-bronchodilatorFEV_1_% predicted, 14) pre-bronchodilator FVC% predicted, 15) FEV_1_/FVC ratio pre-bronchodilator, 16) blood eosinophils, 17) total blood IgE,18) rescue steroid courses taken in last 12 months, 19) beclomethasone dipropionate (BDP) equivalent inhaled corticosteroid dose, 20) age at first assessment, 21) age at onset of symptoms, 22) oral steroid dose, and 23) centre.

**Multiple Imputation**

The multiple imputations work iteratively by selecting a variable as an outcome and then regresses this using the other variables as inputs. The missing values for that variable are predicted using the regression equation informed by the other variables. Other variables are used as outcomes successively using the others as redressers and are repeated until the missing values pass converge tests, [1]. The multiple imputation method has been shown to be less bias than removing the missing data or replacing by the mean [9]. Five datasets are created each with differing values in the missing values for the variables with the values for missing data being selected from the converged solution, see fig E1. Factor and cluster analysis is carried out on each dataset with cluster membership combined for all 5 datasets to find the overall cluster membership for the original data. Further details are found in the sections Factor Analysis and Cluster Model.

**Factor Analysis**

One of the assumptions in the cluster analysis approach used in this paper was that the variables to be used in the cluster model should be independent. However the variables we obtained from the BTS severe asthma registry had a complex correlation structure underpinning them. In order to determine this structure and obtain the best number of independent variables that can represent the severe asthma registry variable structure, factor analysis was implemented.

Factor analysis was carried out in SPSS [4] using the principle components methodology picking the number of factors that were required using the Kaiser criteria that keeps factors that have Eigen values greater than 1 thus keeping only the practical significant factors [10]. The independent factor scores obtained from each factor were used as inputs into the cluster model.

**Cluster Model**

The two-step cluster model in SPSS [5,6] was used for clustering. For the continuous data used the two-step cluster model was in effect a statistical cluster model with multivariate normal clusters assumed. Cluster models from 1 multivariate normal cluster successively to 15 multivariate clusters were carried out. Bayesian Information Criterion model fitting criteria were evaluated at each cluster/model. The Bayesian Information Criterion started large with smaller clusters and then decreased when reaching optimal solutions of clusters and then increased again when over-fitting of clusters occurred.

The number of clusters was determined by first calculating the Bayesian Information Criterion (BIC) [5,6]. The cluster model with the smallest BIC was chosen as the best fitting solution and thus the best fitting number of clusters. The optimal number of clusters for each multiple imputed dataset is as shown in Table E1. The BIC for each multiple imputed dataset for clusters 1 to 15 is shown in Fig E2. Thus five cluster memberships were created one for each of the imputed datasets. These were then needed to be averaged over to obtain a global cluster membership, see “combination of the 5 cluster membership variables”

**Combination of the 5 cluster membership variables.**

To obtain the overall cluster membership of the original data, the 5 cluster memberships have to be combined. This was carried out using the two-step cluster model in SPSS for clustering categorical data. This takes a number of categorical variables, such as cluster membership, and identifies sub-groups within the variables again using the BIC, so we determine overall clusters using the 5 cluster memberships. A summary of the many steps in analysis can be seen in figure E1.

**Classifier**

Classifiers can be derived from cluster membership by determining the best variables that separate the clusters and using the best separating variables statistics for each cluster to determine a predicted unsupervised cluster membership. A classifier was created for the BTS patients using the clinical variables that were used in the cluster analysis The classifier is a function in R to classify the patients into one of the five clusters, based on the specific cluster multivariate parameters that were used in the cluster analysis. Every cluster has a unique multivariate distribution based on a subset of input parameter. A patient is classified as belonging to the cluster with the most likely cluster multivariate characteristics for that patient without the need of the rest of the data to determine a cluster membership. The classifier uses the following variables as inputs, each of the underlying factors is represented in the classifier with extra variables added to improve predictive accuracy, the resulting variables used were;

1. BMI
2. Pre Bronchodilator FEV_1_ percent predicted
3. Blood eosinophil count
4. Age of onset
5. Number of rescue steroid course in previous year
6. BDP inhaled corticosteroid dose
7. And oral steroid dose

Each cluster has a multivariate mean and covariate structure based on the above variables and a probability is computed to determine which is the most likely cluster for each patient based on the likelihood.

Once classified the patients cluster classification was compared with their original cluster classification to determine percentage accuracy at baseline and stability at follow-up. The classifier was applied to a second dataset to determine if the clusters were in similar proportions and had similar characteristics to the original dataset. A p value of <0.05 was taken as the threshold of statistical significance.

Figure E1.Flow chart of data and analysis, Data is in blue rectangles, analysis is in orange rectangles


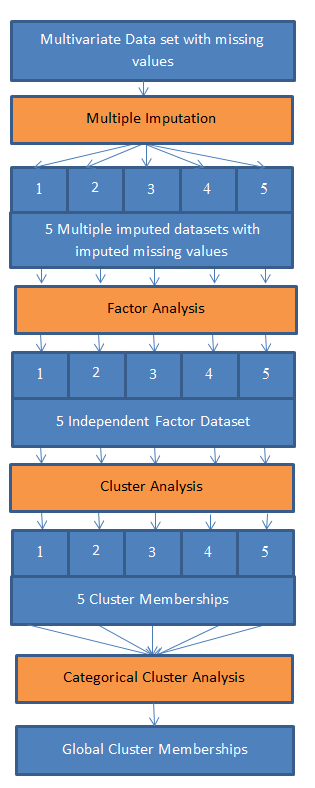


**Figure E2. The BIC for each imputed dataset for models with 1 to 15 clusters. The best fitting cluster model has the smallest BIC. The best fitting cluster model was 5 clusters indicated by the dip in the curves and the arrow pointing to 5.**


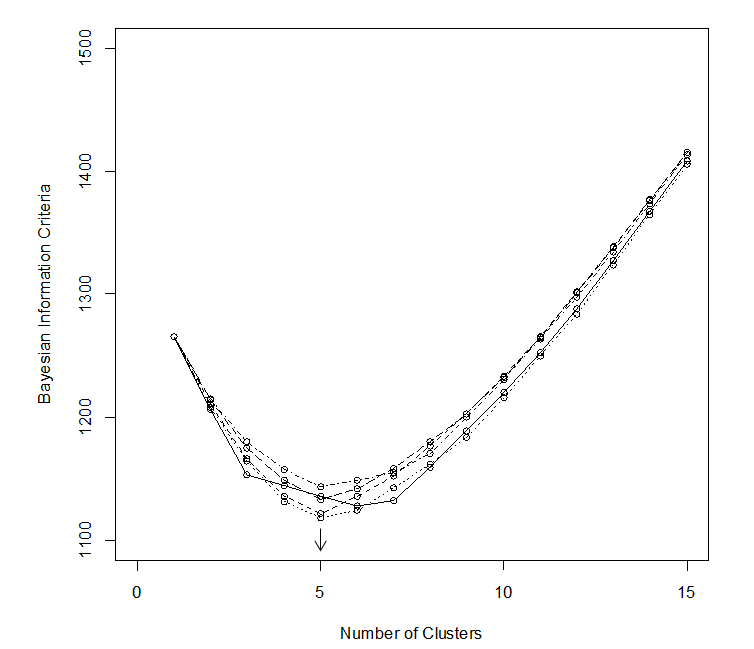


**Legend for Figure E2**

**
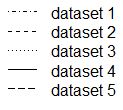
**

**Table E1. The optimal number of clusters for each multiple imputed dataset using BIC**

|  | Dataset 1 | Dataset 2 | Dataset 3 | Dataset 4 | Dataset 5 | overall |
| --- | --- | --- | --- | --- | --- | --- |
| Number of clusters | 5 | 5 | 5 | 6 | 5 | 5 |

**Table E2 Subjects in Clusters by Clinical Centre**

|  | **Cluster 1** | **Cluster 2** | **Cluster 3** | **Cluster 4** | **Cluster 5** |
| --- | --- | --- | --- | --- | --- |
| **First Dataset (p<0.001)** | 117 | 72 | 52 | 54 | 54 |
| Queens University Belfast | 32 | 18 | 11 | 19 | 9 |
| ILH, Leicester | 41 | 18 | 3 | 12 | 18 |
| RBHT, London | 17 | 29 | 11 | 12 | 4 |
| Lung Centre, Manchester | 27 | 7 | 27 | 11 | 23 |
|  |  |  |  |  |  |
| **Second Dataset (p=0.022)** | 90 | 68 | 35 | 32 | 20 |
| Queens University Belfast | 12 | 8 | 4 | 8 | 2 |
| ILH, Leicester | 14 | 11 | 8 | 13 | 1 |
| RBHT, London | 9 | 5 | 2 | 0 | 1 |
| Lung Centre, Manchester | 27 | 18 | 9 | 6 | 9 |
| Stobhill Hospital Glasgow | 4 | 6 | 5 | 3 | 2 |
| Gartnavel General Hospital | 13 | 14 | 2 | 0 | 2 |
| Birmingham | 11 | 6 | 5 | 2 | 3 |

**Table E3 p-values for the clinical characteristics comparison for each variable in each cluster between the original cohort clusters and the classified validation cohort clusters.** Data represented as ‡Mean (SD) tested with t.test, †Median (IQR) tested with Mann-Whitney test, * (%) tested with Chi squared test.

|  |  | Cluster 1  p-value | Cluster 2  p-value | Cluster 3  p-value | Cluster 4  p-value | Cluster 5  p-value |
| --- | --- | --- | --- | --- | --- | --- |
| 1 | Gender, (% Male) (N=349)* | 0.795 | 0.330 | 0.486 | 0.909 | 0.182 |
| 2 | Age At Baseline (years) (n=345) ‡ | 0.854 | 0.254 | 0.752 | 0.908 | 0.240 |
| 3 | Age At Onset Of Symptoms (years) ‡ | 0.126 | 0.026 | 0.913 | 0.164 | 0.744 |
| 4 | BMI (N=342) ‡ | 0.319 | 0.996 | 0.634 | 0.792 | 0.545 |
| 5 | Pack year history (for whole population) (N=349) † | 0.027 | 0.128 | 0.377 | 0.029 | 0.256 |
| 6 | HAD Anxiety Score (N=160) ‡ | 0.271 | 0.304 | 0.001 | 0.770 | 0.780 |
| 7 | HAD Depression Score (N=160) ‡ | 0.949 | 0.532 | 0.025 | 0.179 | 0.188 |
| 8 | Atopy (% Yes) (N=346)* | 0.874 | 0.484 | 0.05 | 0.229 | 0.101 |
| 9 | Total IgE blood count , kU/l (N=319) ‡ | 0.041 | 0.086 | 0.128 | 0.201 | 0.105 |
| 10 | Perennial rhinitis (% yes) (N=340)* | 0.030 | 0.468 | 0.019 | 0.285 | 0.245 |
| 11 | Seasonal rhinitis (% yes) (N=340)* | 0.362 | 0.052 | 0.336 | 0.186 | 0.182 |
| 12 | Eczema (% yes) (N=341)* | 0.764 | 0.309 | 0.475 | 0.212 | 0.245 |
| 13 | Polyps (% yes) (N=340)* | 0.648 | 0.444 | 0.163 | 0.869 | 0.244 |
| 14 | Prior nasal surgery (% yes) (N=340) * | 0.420 | 0.390 | 0.305 | 0.536 | 0.191 |
| 15 | Reflux history (% yes) (N=340)* | 0.099 | 0.033 | 0.001 | 0.024 | 0.216 |
| 17 | Oral steroid dose (mg) (N=342) † | 0.227 | 0.954 | 0.100 | 0.055 | 0.365 |
| 18 | BDP equivalent inhaled steroid dose (mcg) (N=333) ‡ | 0.586 | 0.400 | <0.001 | 0.554 | 0.237 |
| 19 | Blood Eosinophil count 10^9^/l (N=327) † | 0.704 | 0.153 | 0.075 | 0.288 | 0.648 |
| 20 | Rescue steroid courses in last year (n) (N=322) † | 0.950 | 0.001 | 0.011 | 0.203 | 0.989 |
| 21 | Hospital Admission last year (n) (N=344) ‡ | 0.08 | 0.443 | 0.566 | 0.602 | 0.687 |
| 22 | ITU admissions in last year (N=345) ‡ | 0.289 | 0.001 | 0.001 | 0.001 | 0.047 |
| 23 | ITU admission ever (% yes) (N=345)* | 0.115 | 0.001 | 0.001 | 0.001 | 0.083 |
| 24 | Pre-bronchodilator FEV_1_ (L) (N=338) ‡ | 0.020 | 0.409 | 0.138 | 0.881 | 0.868 |
| 25 | Pre-bronchodilator FEV_1_ Predicted (%) (N=330) ‡ | 0.002 | 0.932 | 0.023 | 0.434 | 0.634 |
| 26 | FEV_1_/FVC Pre Bronchodilator (%) (N=329) ‡ | 0.132 | 0.021 | 0.834 | 0.086 | 0.310 |
| 27 | Post-bronchodilator FEV_1_ response (%) (N=239) ‡ | 0.149 | 0.039 | 0.381 | 0.526 | 0.211 |

**References**

[1] Yu-Sung Su, Andrew Gelman, Jennifer Hill, Masanao Yajima (2011).Multiple Imputation with Diagnostics (mi) in R: Opening Windows into the Black Box. Journal of Statistical Software, 45(2), 1-31. URL http://www.jstatsoft.org/v45/i02/.

[2] R Development Core Team (2012). R: A language and environment for

statistical computing. R Foundation for Statistical Computing,

Vienna, Austria. ISBN 3-900051-07-0, URL <http://www.R-project.org/>.

[3] Andrew Gelman and Jennifer Hill. (2007). Data Analysis Using Regression and Multilevel/Hierarchical Models. Cambridge University Press.

[4] IBM Corp. Released 2012. IBM SPSS Statistics for Windows, Version 21.0. Armonk, NY: IBM Corp.

[5] Zhang, T., R. Ramakrishnon, and M. Livny. 1996. BIRCH: An efficient data clustering method for very large databases. In: Proceedings of the ACM SIGMOD Conference on Management of Data. Montreal, Canada: ACM.

[6] Chiu, T., D. Fang, J. Chen, Y. Wang, and C. Jeris. 2001. A Robust and Scalable Clustering Algorithm for Mixed Type Attributes in Large Database Environment. In: Proceedings of the seventh ACM SIGKDD international conference on knowledge discovery and data mining. San Francisco, CA: ACM.

[7] IBM Corp. Released 2012. IBM SPSS Statistics for Windows, Version 21.0. Armonk, NY: IBM Corp.

[8] Schwarz, Gideon E. (1978). "Estimating the dimension of a model". Annals of Statistics 6 (2): 461–464

[9] Little RJA, Rubin DB. Statistical Analysis with Missing Data, book. 200

[10] Anna B, Costello and Jason W. Osborne (2005). Best Practices in Exploratory factor Analysis: Four Recommendations for Getting the Most From Your Analysis. Volume 10. No
